# Concerning the vitamin D reference range: pre-analytical and analytical variability of vitamin D measurement

**DOI:** 10.11613/BM.2017.030501

**Published:** 2017-08-28

**Authors:** Davide Ferrari, Giovanni Lombardi, Giuseppe Banfi

**Affiliations:** 1 Department of Biosciences, University of Parma, Parma, Italy.; 2 Laboratory of Experimental Biochemistry and Molecular Biology, IRCCS Istituto Ortopedico Galeazzi, Milano, Italy; 3 Vita-Salute San Raffaele University, Milano, Italy

**Keywords:** vitamin D, biological variability, analytical variability, standardization, DEQAS

## Abstract

Unlike other vitamins, the vitamin D concentration in blood varies cyclically over the course of the year in relation to genetic (gender, ethnicity, polymorphisms) and environmental factors (sunlight exposure, diet, food-related or direct vitamin D supplementation, skin pigmentation). Although the major diagnostics manufacturers have recently developed improved automated 25-hydroxy vitamin D immunoassays, the intra- and inter-laboratory variability is still high (especially at low vitamin D concentrations) which might lead to incorrect vitamin D deficiency/insufficiency diagnosis. Moreover, despite recent efforts to standardize the assay and minimize its variability, the current bias for measured vitamin D concentrations is often still above the desirable ± 10% criterion. Because the implications of low vitamin D concentrations in non-skeletal diseases are still partially unknown, international guideline recommendations for establishing meaningful ranges, at any time over the course of the year, irrespective not only of environmental and personal factors but also of instrumental variability, are needed. In this review, we discuss the main factors that influence the variability of vitamin D concentrations and whether a centile curve, individually calculated by a theoretical equation considering such factors, might be better suited than a fixed limit to assess abnormal vitamin D concentrations in otherwise healthy subjects. Vitamin D reference ranges during pregnancy, childhood, or diagnosed illnesses, which merit separate discussion, are beyond the scope of this review.

## Introduction

The term vitamin D refers to a group of related steroid hormones involved in several physiological processes centred on the maintenance of calcium homeostasis, but also of phosphate, iron, and zinc, through its binding to the vitamin D receptor (VDR) (*1*). After binding the high-affinity vitamin D metabolite 1,25-dihydroxyvitamin, the receptor undergoes homodimerization and heterodimerization to a retinoic acid X receptor (RXR) (*2*). These complexes recognize specific DNA sequences that regulate the transcription of genes encoding proteins that mediate calcium and skeletal metabolism: osteocalcin, osteopontin (SPP1) and bone sialoprotein (BSP) are involved in the mineralization of the bony extracellular matrix (ECM); membrane calcium channel TRPV6 is involved in intestinal calcium absorption; parathyroid hormone (PTH) and the PTH-related protein (PTHrp) are involved in calcium homeostasis and vitamin D activation; receptor antagonist of nuclear factor kappa-B ligand (RANKL) is involved in osteoblast-osteoclast cross-talk in bone as well as in the immune regulation of osteoclastogenesis; low-density lipoprotein receptor-related protein 5 (LRP5) is involved in Wnt signalling in bone and in other Wnt-dependent tissues; cystathionine-β-synthase (CBS) catalyses the first step of transsulfuration of L-homocysteine in L-cystathionine; as well as many others (*3*-*5*). Vitamin D also increases intestinal calcium absorption by inducing the expression of calbindin, a calcium-binding protein that participates in calcium transport across the cell (*2*). Vitamin D is also involved in the homeostasis of other ions like iron, manganese and zinc by regulating the expression of the SLC39A2 gene which plays an important role in iron homeostasis and the SLC30A10 gene which encodes the metal transporter ZnT10 (*6*-*8*).

**Figure 1:**
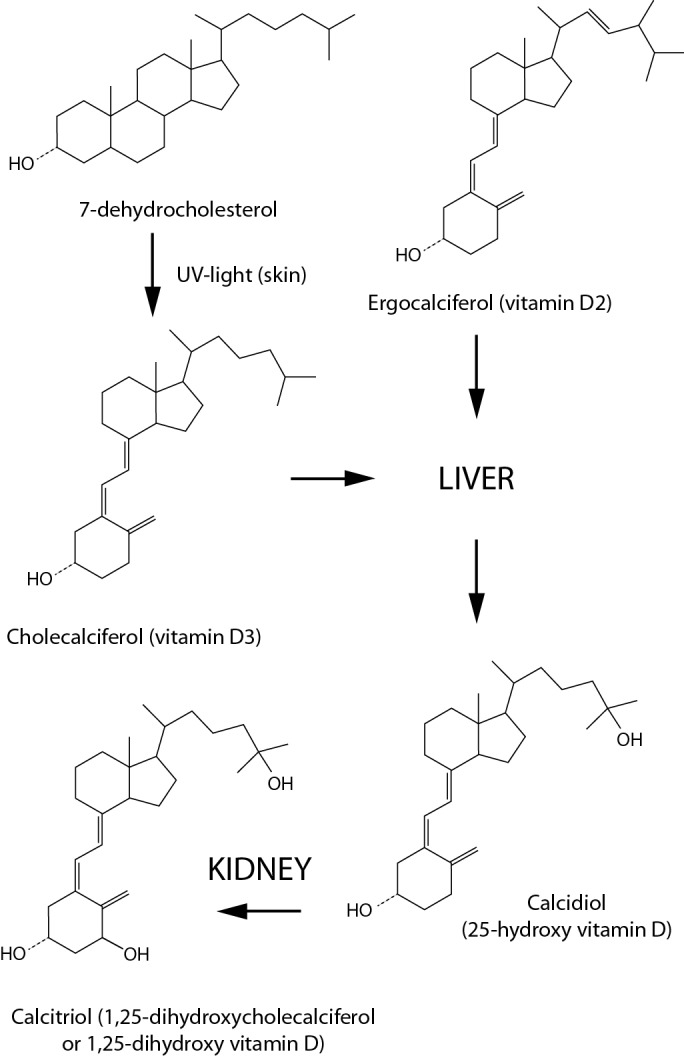
Vitamin D synthesis. 7-dehydrocholesterol (provitamin D) photoreacts in the skin to produce cholecalciferol (vitamin D3). Ergocalciferol (introduced with diet) and cholecalciferol are transformed in calcidiol, by the enzymes present in the liver. The final hydroxylation step exerted by the enzyme CYP27B1, to produce the active form of vitamin D (calcitriol), occurs primarily in the kidney.

There are two forms of vitamin D: vitamin D3 and vitamin D2 (Figure 1). Upon cutaneous exposure to ultraviolet B (UVB) radiation, cholecalciferol (vitamin D3) is synthesized from the photochemical ring-opening and subsequent thermal isomerization of the precursor 7-dehydrocholesterol (provitamin D) by endogenous synthesis (*9*). Vitamin D3 is hydroxylated to form 25-hydroxy vitamin D3 (25(OH)D, calcidiol), the major circulating human vitamin D metabolite, which is then catalysed in the liver by the enzyme 25-hydroxylase (CYP2R1) (*10*). Further hydroxylation activated by the enzyme 1-α-hydrolase occurs in the kidney to produce the biologic active form 1,25-dihydroxyvitamin D3 (1,25(OH)_2_D3, calcitriol) (*10*). Vitamin D2 (ergocalciferol), which accounts for a smaller amount, is of exogenous origin and derives from dietary sources such as plants or fish. Like vitamin D3, vitamin D2 is metabolized in the liver and the kidneys to form 1,25-dihydroxyvitamin D2 (1,25(OH)_2_D2) (*10*, *11*). The abundance of 25(OH)D and 1,25(OH)_2_D is enzymatically regulated by the enzyme CYP24A1 which, by adding a hydroxyl group in position 24, lowers the concentrations of both metabolites (*12*). Vitamin D deficiency can cause rickets in infants and osteomalacia in adults, increasing the risk of osteoporotic fractures (*13*). Since UVB radiation is necessary to synthesize cholecalciferol, vitamin D deficiency in populations living at high latitudes is common especially in winter. However, vitamin D deficiency has become increasingly common also among populations living at lower latitudes owing to the changes brought about by the adoption of modern lifestyle habits (*e.g.*, less outdoor activity, greater usage of sunscreens) often associated with not meeting daily UVB requirements (*14*-*16*). Physical activity seems to be another important factor in the determination of vitamin D concentrations, as documented by the surprisingly high prevalence of vitamin D insufficiency, and even deficiency, in professional athletes regardless of amounts of the sunlight exposure during indoor or outdoor activities (*17*-*19*). Because vitamin D plays a vital role in bone health, inflammation and immunity, skeletal muscle contraction, neuromuscular communication, and cardiovascular function, it is plausible that a suboptimal vitamin D status increases the risk of muscular overuse–related symptoms and inflammatory disease (*20*). Other factors that may impair the synthesis of active D-metabolites (25-(OH)D, 1,25-(OH)_2_D and 24,25-(OH)_2_D) in athletes include skin pigmentation, early- or late-day training, indoor training, geographic location, and extensive sunscreen usage (*17*).

At present, serum total 25(OH)D is considered the best biomarker for assessing vitamin D status (*21*). It is measured by summing the 25-hydroxylated form of the exogenous D2 and the endogenous D3, also called 25(OH)D2 and 25(OH)D3, respectively, both of which have the same biological importance. Although 25(OH)D is not the active metabolite, it is characterized by a longer circulating half-life than 1,25-(OH)_2_D and it is not dependent on PTH which, instead, controls the enzymatic hydroxylation at C1 (*17*). From an analytical point of view, 25(OH)D2 and 25(OH)D3 can be discriminated by chromatographic techniques or mass spectrometry, whereas full or partial cross-reactivity is observed when using immunoassay-based methods. Importantly, unless a patient is taking vitamin D supplementation, 25(OH)D2 usually accounts for less than 5% of total 25(OH)D (*22*, *23*). Based on international measurement standards, serum total 25(OH)D concentration must be reported in nanomoles per liter (nmol/L); however, the use of the unit nanograms per milliliter (ng/mL) is very common, especially in the United States. A 2.6 multiplication factor can be used to convert ng/mL into nmol/L (*24*).

In the bloodstream, most 25(OH)D is bound to vitamin D binding proteins (VDBP) and only a small amount circulates in its free active form and, hence, is able to bind to the VDR (*25*). Since so many environmental and genetic factors can influence vitamin D synthesis, defining a reliable desirable range remains challenging. Furthermore, because of its hydrophobic nature, its high affinity for VDBP, and analytical difficulties, which will be discussed below, 25(OH)D has been defined as a “difficult analyte” (*26*-*28*). Accordingly, the number of publications on how to measure vitamin D has increased in the past decade. To date, only Australia uses a reference range that takes seasonal variation into account (*29*). There is no agreement on the normal ranges for serum total 25(OH)D: the U.S. Institute of Medicine (IOM) suggested a minimal concentration of 52 nmol/L, while the Endocrine Society (ES) suggested a minimal concentration 78 nmol/L (*13*, *30*, *31*). Having such diverse cut-off levels will obviously affect patient categorization.

In this review we discuss the environmental, genetic, and instrumental factors that may influence the measured concentrations of total 25(OH)D and whether a variable range might be more suited than a fixed limit to asses abnormal vitamin D concentrations.

## Pre-analytical variability

### Seasonal effect

Because of its biosynthetic pathway, vitamin D concentration is highly dependent on UVB radiation dose (Figure 1) and varies seasonally at latitudes distant from the equator (*32*). In general, the maximum amount of total serum 25(OH)D in populations living in the northern hemisphere is higher in summer and autumn and lower in winter and spring (*16*, *33*-*35*). O’Neil *et al.* showed that the “vitamin D winter”, defined as the time of year when UVB doses are insufficient to promote vitamin D synthesis, lasts for up to 8 months at latitudes between 60 and 70° N, 5 to 6 months at latitudes between 51 and 59° N, and 2 months or does not occur at all at latitudes between 35 and 40° N (*33*). Krzywanski *et al.* published interesting data about the 25(OH)D concentration in a group of Polish athletes divided into two groups according to whether they were engaged in outdoor sports (OUTD) or indoor sports (IND) (*16*). Additional subgroups were created by dividing the athletes who trained during winter months in countries at lower latitudes like South Africa or Tenerife (SUN) and those who, having inadequate vitamin D status (< 78 nmol/L), were supplemented orally (SUPL). The results showed that the mean value during the course of the year in the IND group was always below the 78 nmol/L (minimal concentration suggested by the ES), whereas the OUT group had mean concentrations of 94 ± 3 nmol/L, slightly above the minimal concentration, but only in the summer. Furthermore, during the winter, both the SUN and the SUPL group had higher 25(OH)D than the OUT group by 85% (~ 120 nmol/L) and 45% (~ 100 nmol/L), respectively, bringing their vitamin D concentrations above the minimal concentration (*16*). Comparably, we have recently demonstrated that the prevalence of vitamin D insufficiency is high in Italian soccer players, despite the latitude, and that 25(OH)D concentrations follow the classical circannual rhythm regardless of the effort spent (*36*).

Latitude-related lower UVB availability does not always correspond to an increased prevalence of vitamin D deficiency, as shown in the study by O’Neill *et al.* (*33*). Among adults living in northern European countries like Iceland and Norway, there were fewer cases (2 - 9%) of vitamin D deficiency in winter as compared to their counterparts living in middle European countries like Ireland (23%). The probable reason is dietary habits: in Norway and Iceland the traditional diet includes large use of fatty fish and cod liver oil and consumption of other vitamin D supplements is more common (*37*, *38*). At lower latitudes, in contrast to the recommendations recently published by the European Food Safety Authority (EFSA) on dietary reference values for vitamin D, supplementation is still uncommon (*39*). This hypothesis is further substantiated by the fact that Norwegian adolescents (aged 13 years), who follow less traditional dietary habits, had, on average, a vitamin D concentrations 26 nmol/L lower than adults (*33*). Even more surprisingly were the results by Katrinaki *et al.* obtained on the population of Crete. Although the UVB availability allows for basically no vitamin D winter, the mean 25(OH)D concentrations were below the deficiency cut-off level of 52 nmol/L for both males and females (50.7 ± 26 and 46.8 ± 24 nmol/L, respectively) (*40*). This was mainly explained by the fact that vitamin D supplementation is seldom used in Greece (either direct or food-related) and by the islanders’ darker skin pigmentation which shields them against UVB radiation. We might also speculate that people living in very hot climates may prefer to spend more time indoors than their counterparts living at higher latitudes where summers are generally cooler by comparison (*41*).

Due to their increasing popularity, a particular mention should be done for vegetarian and vegan diets. Several studies all over the world have shown that vegetarians and vegans are particularly exposed to vitamin D insufficiency and deficiency (*42*, *43*). Also lactovegetarians and lactoovovegetarians show lower concentrations of 25(OH)D compared to non-vegetarians (*44*, *45*). Vegetarians and vegans, hence, are recommended to supplement their vitamin D intake. However, since vitamin D3 is derived from animal sources, vegans who, instead, prefer the plant-derived vitamin D2 avoid it. Actually, there are controversies about the equivalence of these two forms of vitamin D (*23*, *46*-*48*).

In contrast to vitamin D deficiency, toxic concentrations of total 25(OH)D may cause hypercalcemia (*49*). Although hypercalcemia does not normally occur at concentrations below 260 nmol/L, we could exploit the high dependence of vitamin D synthesis on UVB radiation to define a safe upper limit based on healthy subjects having maximal UVB exposure. For instance, in native populations living at equatorial latitudes 25(OH)D concentrations range between 60 nmol/L and 177 nmol/L (*27*). Similarly, healthy subjects not living at the equator but having very high sunlight exposure (like surfers, tanners and outdoor workers) show 25(OH)D concentrations ranging between 73 nmol/L and 177 nmol/L. Based on these data, we can assume that 25(OH)D concentrations of 170-180 nmol/L could be considered as a safe range for healthy people who are often exposed to high UVB doses (*27*).

From a diagnostic point of view, when measuring 25(OH)D concentration, the circannual rhythm of this metabolite should be considered to avoid repercussions on the diagnosis of vitamin D insufficiency or deficiency. For example, in a patient with summer/autumn values slightly above the minimal threshold suggested by the IOM or the ES, the winter/spring values will probably fall below the desirable range. However, except for the Australian guidelines, the current reference ranges for this metabolite do not include seasonal variation; instead, a single measurement taken at any time of the year is used to extrapolate the patient’s vitamin D status throughout the year (*29*).

### Genetics

Genome-wide association studies have shown that single nucleotide polymorphisms (SNP) in the vitamin D pathway genes (DHCR7, CYP2R1, CYP3A4, CYP27A1, DBP, LRP2, CUB, CYP27B1, CYP24A1, VDR, and RXRA) influence vitamin D status (*50*-*52*). While the vitamin D concentration is tightly regulated by the expression level of the enzymes involved in its activation and inactivation pathways, such factors account for approximately 5% of total 25(OH)D variability (*10*, *50*). Consequently, SNPs in the CYP2R1 and DHCR7 genes encoding the enzymes involved in the first steps of 25(OH)D biosynthesis are of limited importance, yet they have been consistently shown to alter vitamin D status. The most studied SNPs are rs10741657 G>A in the CYP2R1 gene which is associated with higher circulating concentrations of 25(OH)D, and rs12785878 G>T in the DHCR7 gene which is, instead, associated with lower circulating 25(OH)D (*50*, *53*-*56*). In contrast, circulating concentrations of VDBP can sequestrate up to 90% of total 25(OH)D (*57*). Because the bioactive form of vitamin D is its free circulating form, it has been proposed that high levels of VDBP may lower the concentration of free 25(OH)D and therefore inhibit its physiological role. As expected, mutations in genes encoding VDBP are the most widely investigated, and a number of SNPs have been consistently associated with altered concentrations of vitamin D. Among others, the most extensively studied are the rs4588 and rs7041 genetic polymorphisms which are associated with increased risk of vitamin D deficiency particularly in East Asian population (*57*-*62*). Variants of VDBP differ markedly between racial groups. For instance, rs1155563 and rs2298849 are associated with lower vitamin D concentrations in African population whereas SNP in the rs17467825, also lowering the concentration of vitamin D, are more frequently found in European population respectively (*51*, *63*-*65*). In general, black Americans have lower concentrations of 25(OH)D and VDBP than their white counterparts, despite similar concentrations of bioavailable (free) 25(OH)D (*66*). The interdependency between VDBP and 25(OH)D concentrations does not seem to have a linear relationship but rather becomes significant at low concentrations of 25(OH)D. For instance, high VDBP concentrations have been correlated with the risk of colorectal cancer (CRC) only in patients with low 25(OH)D concentrations, suggesting that in individuals with normal 25(OH)D concentrations the VDBP concentration does not significantly influence the bioavailable vitamin D (*25*). In other words, the higher the VDBP concentrations are, the greater is the amount of bound 25(OH)D; this would lead to reduced concentrations of free and bioavailable 25(OH)D. It follows that the impact of high VDBP concentrations is more pronounced in conditions of low total 25(OH)D.

### Age, gender, BMI and ethnicity

A recent study by Vuistiner *et al.* reported that gender has little influence on 25(OH)D concentrations (*34*). Similarly, the effect of age was negligible in adults. A separate discussion, which is beyond the scope of this review, concerns pregnant women in which low 25(OH)D concentrations are observed worldwide, children, in whom vitamin D deficiency can cause rickets and might be a risk factor for future chronic diseases, and persons with diagnosed illnesses (*27*, *67*).

Vuistiner *et al.* also showed that body-mass index (BMI) is inversely correlated to vitamin D concentrations. This is mostly due to a decrease in time spent in outdoor activities, inadequate diet, and the sequestration of vitamin D by subcutaneous fat (*68*). As mentioned above, although many of the published studies have been conducted on white people, 25(OH)D concentrations may differ substantially depending on skin pigmentation. Since the main source of vitamin D is exposure to sunlight, dark skin shields against UVB radiation, increasing the risk for vitamin D deficiency (*69*). For instance, populations of African origin differ in skin darkness primarily due to the ratio of eumelanin to pheomelanin (*70*). This difference has genetically evolved because of the different UVB regimes present in their countries of origin and might pose a higher risk of developing vitamin D deficiency in people of African ethnicity living at higher latitudes (*71*, *72*). Similarly, genetic variants of VDBP can be observed between racial groups (*73*). These factors should be taken into account when measuring total serum 25(OH)D in different ethnic groups, particularly in those living at latitudes greatly different than those of their ancestors (*27*).

## Analytical variability

Precise measurement of vitamin D concentrations is difficult, and large variations exist between different assay methodologies. Such variations depend on several factors: different methods of vitamin D extraction, antibody cross-reactivity with epimers and/or other vitamin D metabolites, and presence of isobaric compounds or matrix interferences (*17*, *21*). Table 1 lists the assays most commonly used in clinical laboratories. The assays can be divided in two main categories: 1) assays based on a chromatographic separation step, the most popular of which are liquid chromatography-mass spectrometry (LC/MS) or liquid chromatography-tandem mass spectrometry (LC-MS/MS), and 2) non-chromatographic methods based on antibody or protein binding, such as immunoassays. The chromatography-based assays are more consistent and accurate than the antibody-based methods. Since the above mentioned methods are usually based on achiral chromatographic techniques, they cannot distinguish between 25(OH)D3 and its 3-epimer (which concentrations are high in infants but represent only 6% of total measured 25(OH)D in adults), or other isobaric compounds such as 7-α-hydroxy-4-cholesten-3-one (an endogenous precursor of bile acids) resulting in a slight overestimation of vitamin concentrations (*74*). This problem can be overcome, however, by appropriate (*i.e.* chiral phase) chromatographic separation (*75*).

**Table 1:** Currently used vitamin D methods

**Method**	**Proportion, %**
Radio immunoassay	2
Manual immunoassay	9
Automated immunoassay	69
LC-MS/MS	15
Other	5
LC-MS/MS - liquid chromatography-tandem mass spectrometry.	

In order to promote laboratory measurement standardization and reduce variability of vitamin D measurement, in 2010 the Office of Dietary Supplements (ODS) of the U.S. National Institutes of Health (NIH) organized the Vitamin D Standardization Programme (VDSP) (*76*). This Programme also involves the National Institute for Standards and Technology (NIST), the Centers for Disease Control and Prevention (CDC), the Vitamin D External Quality Assessment Scheme (DEQAS), the College of American Pathologists (CAP), the American Association for Clinical Chemistry (AACC), and the International Federation of Clinical Chemistry and Laboratory Medicine (IFCC). The VDSP is now monitoring performance, accrediting laboratories engaged in vitamin D assays, and developing a standard reference measurement procedure (SRMP) system which consists of a set of components and procedure that can be used to calibrate the clinical laboratory instrumentation similarly to the Standard Reference Materials (SRMs) previously produced by the NIST (*24*, *77*). On the same line, the CDC provides a vitamin D standardization certification programme and publishes a list of those manufacturers/laboratories that have successfully passed the performance criterion (± 5% mean bias and overall imprecision of < 10% over the concentration range of 22 - 275 nmol/L for total 25(OH)D). An additional resource to standardize small clinical and research laboratories, as an alternative to the CDC programme, which is expensive and more suited for manufacturers and large laboratories, are the accuracy‐based performance testing (PT) programs offered by the CAP and the DEQAS. The latter distributes quarterly five serum samples with 25(OH)D concentrations previously determined by the NIST to registered laboratories. The laboratories then return their results for quality assessment by the DEQAS (*78*).

Although much effort has gone in improving accuracy and precision in 25(OH)D measurements, two recent studies have highlighted substantial within‐assay and between‐assay variability across different commercially available instrumentations (*26*, *27*). Both studies compared the results obtained by LC-MS with 5 automated chemiluminescent immunoassays (CLIA) from different manufacturers (Abbott Diagnostics, Diasorin, IDS, Roche Diagnostics, and Siemens) and found that the mass-spectrometry instrumentation has the best performance with a bias < 10%, even at a concentration as low as 5.2 nmol/L. In contrast, most immunoassays had a bias greater than ± 15%, and as large as 30% in some cases. Only the Liaison instrument from Diasorin showed a bias of only 6.4%, which is comparable with that of LC-MS methods. However, the immunoassay biases increased dramatically at low vitamin D concentrations (< 21 nmol/L): the bias increased up to 35% for the Liason, whereas for the other instruments it exceeded 100% (*26*).

The large discrepancies between LC-MS methods and immunoassays, but also among different immunoassay methods, is mainly due to the differences in cross-reactivity with various vitamin D metabolites, which accounts for a significant proportion of total 25(OH)D (*27*, *79*). Although immunoassays do not detect 3-epi-25(OH)D3, generating specific antibodies against small antigenic molecules such as 25(OH)D is challenging, and cross-reactivity with 24,25(OH)_2_D3 (a product of the vitamin D catabolic pathway that can be present at concentrations of up to 13 nmol/L) and other metabolites of the vitamin D pathway is common (*10*, *80*). While some immunoassays cannot detect 25(OH)D2, those that can are unable to distinguish between 25(OH)D2 and 25(OH)D3, making it difficult to determine abnormalities. Furthermore, the strongly hydrophobic 25(OH)D is largely bound to VDBPs in blood which compete with the antibody in assays where 25(OH)D and VDBP are not completely separated; manual extraction can overcome this problem but increases imprecision (*26*).

**Figure 2:**
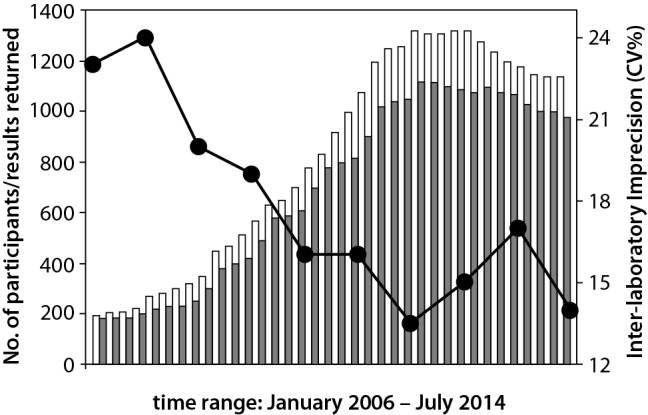
Relationship between the mean inter-laboratory imprecision (line and dotted plot) and the number of DEQAS (25-Hydroxyvitamin D measurements) registered laboratories (white bars). Gray bars indicates the numbers of returned results. With permission from DEQAS.

**Figure 3:**
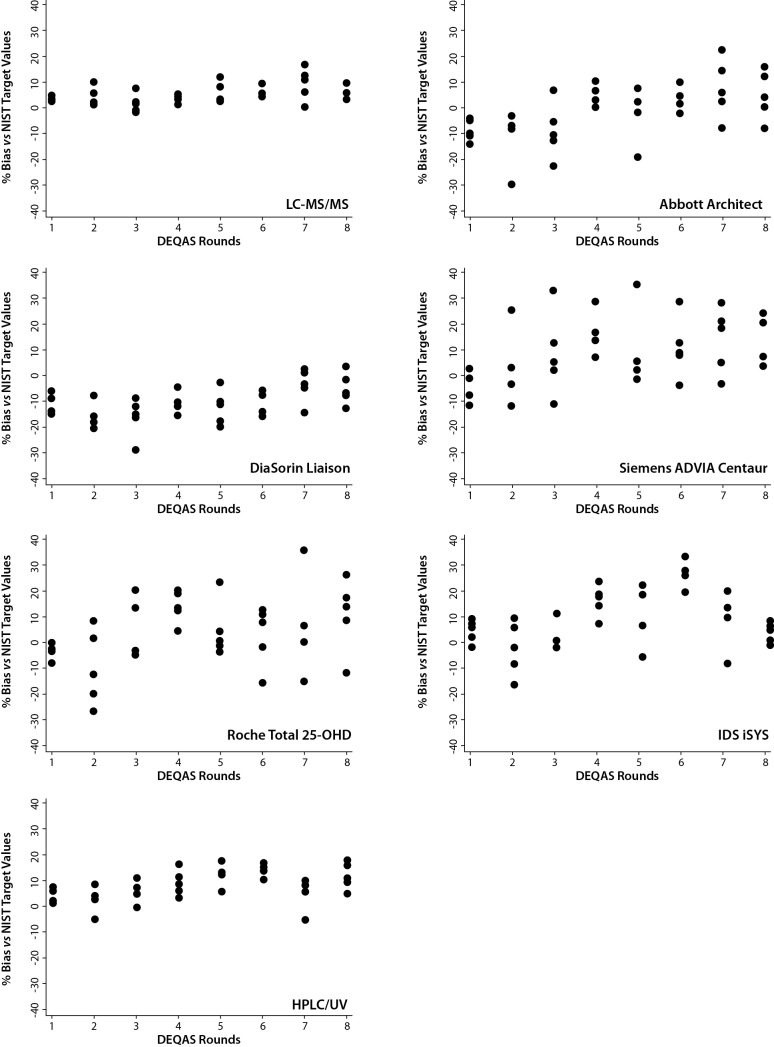
Changes in % bias from the NIST assigned values for each of the major 25(OH)D methods. 40 samples were distributed quarterly (8 rounds, 5 samples at a time) between October 2012 and July 2014 (*54*). With permission from DEQAS.

The DEQAS noted a substantial improvement in the accuracy and precision of vitamin D measurements after the release of the NIST SRM reference in 2008 (*81*). Figure 2 shows how the inter-laboratory imprecision has dropped dramatically since then and nicely correlates with the rise in the number of participants in the Assessment Scheme (78). As illustrated in Figure 3, and consistent with the studies by Farrell and Fuleihan discussed above, the mean bias (from the NIST-assigned target values) for a total of 50 samples distributed between 2012 and 2014 has not changed much during this 3-year time period. However, while the LC-MS/MS and HPLC/UV methods have a lower bias (within ± 15%), and except for the Diasorin Liaison which has a bias comparable with the LC-MS methods, the other tested immunoassay instruments have biases as high as ± 30% (*78*). To overcome the problem associated with substantial inter-laboratory variability, the VDSP conducted a retrospective standardization by analysing stored samples available from two national studies: The Third National Health and Nutrition Examination Survey (NHANES III, 1988–1994) and the German Health Interview and Examination Survey for Children and Adolescents (KIGGS, 2003–2006) (*82*, *83*). The retrospective standardization showed that the vitamin D values were overestimated in NHANES III and underestimated in KIGGS. When properly applied, the assay standardization proposed by the VDSP can greatly improve the interpretation of research data. Nonetheless, we are still far from having a worldwide vitamin D assay standardization that is capable to provide reliable measurements with a bias within the desirable ± 5% range. Table 2 lists the main advantages and limitations of MS-based methods and immunoassays for vitamin D measurement.

**Table 2:** Advantages and limitations of MS-based methods and immunoassays for vitamin D measurement

**Method**	**Advantages**	**Limitations**
**MS-based**	high sample volume (LC-MS) availability of reference measurement procedures separation of vitamin D metabolites allows the inclusion of stable isotope-labelled standards	not intended for routine clinical samples analysis not fully automated complex and expensive instrumentation no distinction between epimers
**Immunoassays**	high sensitivity reduced sample volume fully automated	cross-reactivity with other vitamin D metabolites (*e.g.*, D2 vs. D3) measurement of free 25(OH)D requires VDBP displacement (proprietary methods)
LC-MS – liquid chromatography-mass spectrometry. VDBP - vitamin D binding proteins.		

## Reference ranges

Measuring 1,25-(OH)_2_D in serum is more difficult than measuring its precursor 25(OH)D due to its peculiar properties: highly lipophilic, highly instable, presence at picomolar concentrations within the circulation, below the detection limits of direct UV or MS methods. Moreover, the immunoassay-based determination of 1,25(OH)_2_D is affected by the cross-reactivity of antibodies with other vitamin D metabolites thus requiring chromatographic sample pre-purification processes, with extensive manipulation of the sample (*84*). Serum 25(OH)D is thus considered the best indicator of the vitamin D status even because, contrarily to 1,25(OH)_2_D, it is not dependent on PTH and directly reflect the entity of the vitamin D stores (*18*). Current guidelines from scientific bodies recommend the measurement of 25-hydroxy vitamin D (25(OH)D) in blood as the preferred test, however, because the total serum 25(OH)D concentration has several subject-specific and environmental-dependent sources of variability, the adoption of a fixed desirable range is inappropriate. The main biological source of variability arises from the tight dependence between UVB exposure and 25(OH)D concentration, resulting in a wide variability of vitamin D concentrations over the course of the year. This should be considered when measuring vitamin D in individual patients, because summer values slightly above the desirable range of 52 nmol/L, as suggested by the IOM, will probably fall below such concentrations in the wintertime. Ideally, subject-specific factors related to UVB availability (*e.g.,* outdoor activity, apparel, skin pigmentation, use of sunscreens, living latitude, and winter holidays at low latitudes) should be taken into account to calculate the desirable range. Other factors to be considered are: ethnicity, BMI, and food-related or direct vitamin D supplementation.

Although the short-term biological variability (6 weeks) for 25(OH)D is less than 7%, its concentrations vary, on average, by 40 nmol/L during the year, with peak changes of up to 105 nmol/L (*34*, *85*). Fohner *et al.* showed that in healthy subjects living at high latitudes more than 50% of the biological variability for vitamin D could be explained by relatively few factors (*e.g.,* age, diet, gender, season of sample collection, BMI, latitude, and genotype), while the remaining variability could be ascribed to vitamin D supplementation (*86*). Similarly, Rees *et al.* analysed a multivariable model and showed that few factors (sex, baseline serum 25(OH)D, adherence to contraceptive pill intake, apparel, physical activity, use of extra vitamin D-containing supplements, and season of blood collection) accounted for 50% of vitamin D variability after cholecalciferol supplementation (*87*). In addition, BMI was associated with baseline serum 25(OH)D but not with its response to supplemental cholecalciferol, and genetic factors did not play a major role, either. Veugelers *et al.* who proposed three different Recommended Dietary Allowances (RDA) for normal weight, overweight, and obese patients, reported a relationship between BMI and vitamin D supplementation (*88*).

**Figure 4:**
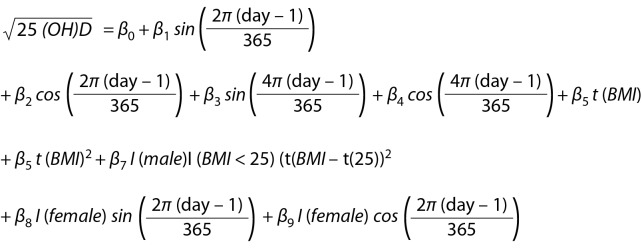
Predictive model proposed by Vuistiner *et al.* (*34*) for 25(OH)D concentrations. The model was set according to the Bayesian Information Criterion (BIC). Body-mass index (BMI) was transformed using an inverse square-root function t(x) = − 1/√x. Model’s coefficients were as follows: β_0_ = − 2.754 (P = 0.295), β_1_ = − 1.077 (P < 0.001), β_2_ = − 0.756 (P < 0.001), β_3_ = 0.188 (P < 0.001), β_4_ = 0.025 (P = 0.368), β_5_ = − 81.08 (P = 0.002), β_6_ = − 165.6 (P = 0.014), β_7_ = − 1174 (P ≤ 0.001), β_8_= 0.218 (P < 0.001), and β_9_ = 0.164 (P = 0.003). The model explained 29% of the variance.

In light of these studies, it appears that the desirable range for vitamin D should be calculated using a validated equation that takes into account the UVB-component, ethnicity, BMI, age, sex, and eventually vitamin D supplementation. Recently, predictive models have been developed that take into account seasonal variability and ethnicity, and seasonal variability and BMI (*34*, *89*). In the former study, O’Neil *et al.* proposed a predictive model (Figure 4) that, by combining the effect of seasonal variability and a component accounting for food-related or direct supplement vitamin D fortification, successfully predicted the measured wintertime 25(OH)D concentration for both white and black Asian minority ethnicity (BAME) population groups (*89*). In another work by Vuistiner *et al.,* the data from more than 7000 people were used to create a population-based model that predicts the centiles of the 25(OH)D distribution by gender, age, BMI, and taking seasonal variation into account. The model can be used to predict future values of an individual over the course of the year based on a measurement made on a given day. The study involved only white Caucasians to avoid the vitamin D variability caused by differences in ethnical skin pigmentation (*34*). When the determinants of vitamin D concentrations are different, as for example, two different ethnicities or very different latitudes, the predictive model has to be modified accordingly. Additionally, predictive models can be implemented by including population-pharmacokinetic models, as described by Ocampo-Pelland *et al.* (*90*). The available mathematical models seems to be more predictive of vitamin D concentrations in healthy subjects whereas for diseased individuals, or in case of specific physiological conditions (*e.g.* pregnancy), the adoption of a unique equation might be inappropriate and, in these cases, more frequent measurements of serum 25(OH)D should be the preferred option.

A separate issue concerns instrumental variability which closely depends on the type of assay used. Although the VDSP is making great effort to reduce the within-assay and between-assay variability, the DEQAS review shows that, except for LC-MS, the bias for the majority of the currently used instrumentations (see Table 1) is still high and is likely to influence treatment decision making (*78*).

## Conclusions

The prevalence of vitamin D deficiency and insufficiency is high, and may possibly increase in the future. Therefore, it is desirable to include assessment of vitamin D in routine examination in order to monitor its concentrations and to follow up eventual supplementation regimens. Provided that accurate 25(OH)D value can be measured, the desirable range should be extrapolated, in individual patients, by an equation considering the time of the year, sun exposure, ethnicity, BMI, the type of assay used and possible intake of vitamin D, that can predict the 25(OH)D centile curve for an healthy subject. The discrepancy between the predicted value and the measured 25(OH)D concentration, at any time of the year, will be then safely used to determine an accurate diagnosis on the patient vitamin D status. If such equations have been developed for otherwise healthy individuals, additional parameters or completely different equations will be needed to assess individual situations like pregnancy, childhood, or diagnosed illnesses.

Although the situation has substantially improved through the efforts of the VDSP, what is still lacking is a general standardization, or at least a harmonization, of methods that provide comparable and, more importantly, less biased results. Ideally, all measurements should be performed using LC-MS; however, this scenario being impracticable, we encourage clinical laboratories to adopt an assay traceable to the gold SRMP as proposed by the VDSP in order to calibrate their new and, if available, old measurements (*91*, *92*).
